# Effect of Physical Exercise Programs Based on Mobile Health and Ecological Momentary Assessment on the Physical and Mental Health, Cognitive Functions, and Social Environment of Adults in Developing Countries: A Systematic Review

**DOI:** 10.3390/medicina60040578

**Published:** 2024-03-31

**Authors:** Alejandro Flores Aniotz, Daniel Reyes-Molina, Igor Cigarroa, Sonia García-Merino, Margarita Rubio Alonso, Margarita Pérez Ruiz, Rafael Zapata-Lamana

**Affiliations:** 1 Programa Vida Saludable, Universidad de Talca, Talca 3460000, Chile; canoaniotz@gmail.com; 2 Facultad de Ciencias de la Actividad Física y el Deporte y Fisioterapia, Universidad Europea de Madrid, 28670 Madrid, Spain; 3 Escuela de Kinesiología, Facultad de Salud, Universidad Santo Tomás, Los Ángeles 4440000, Chile; danielreyes@udec.cl; 4 Facultad de Ciencias Sociales, Universidad de Concepción, Concepción 4070386, Chile; 5 Escuela de Kinesiología, Facultad de Ciencias de la Salud, Universidad Católica Silva Henríquez, Santiago 8240000, Chile; 6 Facultad de Ciencias de la Salud, Universidad Arturo Prat, Victoria 4720000, Chile; 7 Facultad de Ciencias de la Salud, Universidad Francisco de Vitoria, 28223 Pozuelo, Spain; sonia.garciamerino@ufv.es; 8 Departamento de Medicina, Facultad de Ciencias Biomédicas y de la Salud, Universidad Europea de Madrid, 28670 Madrid, Spain; margarita.rubio@universidadeuropea.es; 9 Departamento Salud y Rendimiento Humano, Facultad de Ciencias de la Actividad Física y el Deporte Universidad Politécnica, C. de Martín Fierro, 7, Moncloa-Aravaca, 28040 Madrid, Spain; margarita.perez@upm.es; 10 Escuela de Educación, Universidad de Concepción, Los Ángeles 4440000, Chile

**Keywords:** mobile health, momentary ecological assessment, physical exercise, systematic review

## Abstract

*Background and Objectives:* Although there is strong evidence of the positive effects of physical exercise on health, adherence to face-to-face exercise programs in the adult population is low, identifying several barriers that hinder their practice. There is research that demonstrates the viability of physical exercise programs with the use of Mobile Health in Ecological Momentary Assessment (EMA) mode, which contributes to overcoming many reported barriers. To synthesize the methodological characteristics and health effects of physical exercise programs based on mobile health in EMA modality in adults in developing countries. *Materials and Methods:* This systematic review was conducted according to guidelines established by the PRISMA statement in APA PsycArticles and CINAHL databases by EBSCOhost, Cochrane Library, PubMed, and Web of Science for articles published between 2008 and March 2024. *Results:* Telephone counseling on clinical–behavioral factors is believed to reduce morbidity and mortality in developed countries, but this aspect is not explored in developing countries. We included nine randomized controlled trials with a total of 4394 male and female participants aged 18 to 60 years. The interventions were mainly carried out by text messages, lasting between 20 to 80 min per session, 3 to 5 days per week, and most were carried out over 12 months. The interventions on the variables of physical activity, nutrition, and medical assessments showed significant effects, and variables such as quality of life and anthropometric measurements were not significant in most studies. *Conclusions:* This systematic review included studies from different developing countries, the most common diseases being diabetes, overweight, obesity, and hypertension. All the studies used mobile devices as the technology, finding a profile of the adults studied, as well as the characteristics of exercise programs based on mobile health in EMA modality.

## 1. Introduction

The benefits of physical exercise on people’s health have been widely documented over time; in particular, a consensus has been established on its importance as a treatment for various chronic diseases [1]. These benefits extend to physical, metabolic, psychological, cognitive, and general well-being, as well as social integration [2]. In addition, in adults, exercise has been shown to promote cardiovascular health by stimulating myocardial regeneration and reducing age-related loss of muscle mass and strength, a risk factor for nontraditional cardiovascular disease [3].

Even though the health benefits of physical exercise are well known, about 27.5% of adults globally [4] do not meet physical activity recommendations proposed by the World Health Organization (WHO) [5]. This lack of activity is associated with poorer health outcomes, including higher all-cause mortality, type 2 diabetes, and cancer [6,7,8].

Adults with high glucose levels and a Body Mass Index (BMI) outside established ranges have an increased risk of presenting with chronic non-communicable diseases such as hypertension, obesity, type 2 diabetes mellitus, cardiovascular diseases and different types of cancer. WHO (2018) stated “that chronic non-communicable diseases claim the lives of 41 million people each year in the world. Likewise, 15 million adults die every year in low- and middle-income countries” [9].

Different studies have identified that, among adults, physical limitations due to pain and weakness, lack of motivation, lack of time management, lack of money, adequate facilities and spaces, and a way to travel to the place where the exercise is performed are the main perceived barriers to physical exercise [10,11,12]. More recent ones have been related to the method of commuting for exercise, a lack of money, and a lack of adequate facilities and spaces [11].

Lastly, the restriction of mobility caused by the Coronavirus disease (COVID-19) pandemic has forced many to look for different alternatives to engage in physical activity without the need to leave their place of residence. In this context, virtual or remote exercise with the use of technology has been promoted and proposed as a promising alternative in different countries and in people of different ages and with health conditions [13,14].

In this context, studies have been carried out that demonstrate the feasibility of remote physical exercise programs through mobile health (mHealth), which helps to overcome many of the previously mentioned barriers, such as the optimization of travel time and the elimination of geographical restrictions [15]. mHealth refers to the use of mobile devices such as smartphones, tablets, and patient-monitoring devices to facilitate health management [16]. Although mHealth is a common practice in medicine, its emerging application in physical activity and exercise has great potential to combat inactivity in adults and improve health through remotely supervised and monitored exercise programs [17].

In addition to the use of mHealth, the Ecological Momentary Assessment (EMA) is a tool that allows for real-time assessment of contextual variables in natural environments, reducing the limitations of retrospective assessments on daily behavioral and context variables [18]. In relation to evaluation based on memory and observation, it is relevant to emphasize the difference in how experiential information is stored vs. memory; its usefulness when recording it underlies the fact that immediate information is stored in experiential or episodic memory. Instead, beliefs about what has happened about the experiences that occur to us are stored in semantic memory [19]. This makes it a promising tool for understanding exercise-related behavior [20,21].

However, despite advances in the use of mHealth in EMA mode in the promotion of physical activity in adults, there are challenges and limitations that require further research. Many of the studies have been conducted in developed countries, highlighting the need for further research on the usefulness of these interventions in developing countries and vulnerable populations [22]. Another aspect is that there has been no evidence of studies in vulnerable populations [23], and the use of mHealth and EMA has the unique potential to improve program outreach to the populations most in need [24]. Even though technology-mediated exercise is on the rise, there are still many environments in which the Internet is not accessible; therefore, these potential limitations must be considered [25].

Another challenge is the heterogeneity in the methodological aspects of studies using mHealth in EMA mode in practice, which makes comparison difficult due to differences in assessment instruments and sample populations [26,27,28,29,30]. In addition, the following few studies were identified, and of these, most had small sample sizes and very short durations of interventions [23,27,29,31,32]. Last, there are a few studies that look at gender differences [33].

Studies that use mHealth and EMA in exercise for health address different aspects such as mental health, tobacco, alcohol, and cardiometabolic diseases such as diabetes, high blood pressure, and overweight and obesity. However, there is limited evidence from systematic reviews synthesizing the effects of mHealth and EMA physical exercise programs on adult health in developing countries. The research question was: What effect do physical exercise programs based on mobile health and momentary ecological assessment have on the health of adults in developing countries?

Therefore, the aim of this systematic review is to synthesize the methodological characteristics and effects on physical health, nutritional status, and quality of life of physical exercise programs based on mobile health in the Ecological Momentary Assessment mode in adults from developing countries. We hypothesize that physical exercise programs based on mHealth and EMA in adults in developing countries have positive effects on health.

## 2. Materials and Methods

This systematic review was conducted in accordance with the standards set by the PRISMA statement [34]. The PRISMA checklist can be found in the Supplementary Material. Registration number: CRD42022347874.

### 2.1. Search Strategy

The search strategy followed the guidelines of the Peer Review of Electronic Search Strategies (PRESS) [35]. A systematic search of the APA PsycArticles and CINAHL databases was conducted using EBSCOhost, Cochrane Library, PubMed, and Web of Science. The general search phrases, limited by title, abstract, and keywords, was: (“mobile health” OR “mhealth”) (“ecological momentary assessment” OR “ambulatory assessment” OR “mea”) AND (“exercise” OR “physical activity”). The search was conducted in July 2022 and updated until March 2024, and was limited to studies published from January 2008 onwards in English or Spanish.

### 2.2. Study Selection and Eligibility Criteria

All articles that met the search phrase were considered. Then, only those articles that met the following inclusion criteria were selected (Table 1).

### 2.3. Data Extraction

The selected articles were independently reviewed by two reviewers, and any discrepancies were discussed with a third reviewer until an agreement was reached.

Data extraction was carried out in three stages. First, duplicate records obtained from the databases were eliminated using the Zotero 5.0 bibliographic manager. Second, two review authors selected records that met the inclusion criteria based on reading the titles and abstracts of the articles. Third, when decisions could not be made based on the title and abstract alone, the full-text documents were retrieved.

### 2.4. Risk of Bias Assessment Tool

We used the Cochrane Collaboration tool to assess the risk of bias in 9 randomized clinical trials included in this systematic review. The Cochrane Collaboration tool covers six domains of bias: (D1) selection bias, (D2) performance bias, (D3) detection bias, (D4) attrition bias, (D5) reporting bias, and overall) to evaluate the overall risk of bias. Within each domain, evaluations were performed for one or more elements, assigning a judgment of high, low, or unclear risk of bias [38]. Each article was rated by one of the reviewers (I.C), and the results of each article are presented in a traffic light diagram with the synthesis of risk of bias. To graph the result of the risk of bias, the Robvis tool was used [39].

### 2.5. Strategy for Data Synthesis

**Figure 1:**
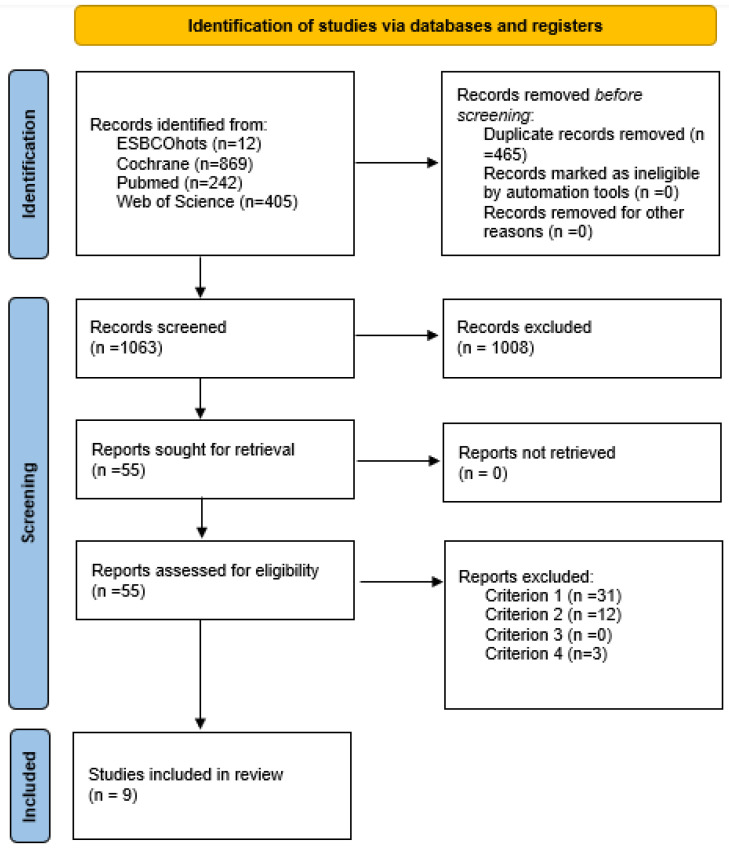
Study selection flowchart according to the PRISMA statement [34].

A narrative synthesis of the main findings of the included articles is provided. The stratification of the selected studies is plotted in Figure 1 using the PRISMA flowchart [34]. The information extracted included: (a) General characteristics of the included studies (Table 2); (b) general characteristics of the interventions of physical exercise based on mHealth and EMA (Table 3); (c) effect of physical exercise based on mHealth and EMA; and instruments used. It contains variables such as physical activity, quality of life, nutrition, anthropometric measurements, and medical evaluations (Table 4: Qualitative tables).

## 3. Results

### 3.1. Selection of Studies

Figure 1 presents the flowchart for systematic reviews proposed by the PRISMA Declaration. A total of 1528 potential articles were identified. Subsequently, after the exclusion of duplicates from the databases, the selection and eligibility criteria were applied. Finally, nine articles were included for the narrative synthesis of this review [40,41,42,43,44,45,46,47,48].

### 3.2. General Characteristics of the Included Studies

Of the nine included, six were carried out in South Asian countries [40,41,42,44,45,47], one in North America [46], and two in South America [43,48]. As for the sample, men, and women with cardiometabolic diseases participated, with diabetes and overweight and obesity being the main diseases. Four studies were also conducted including patients with type 2 diabetes [42,45,46,47], and one was conducted on overweight and obese patients [42]. The studies included both men and women, with a final sample of 4394 participants, of which 1981 were women.

Participants’ adherence to exercise interventions ranged from 40% in one study [48] to 89% in the study with the highest adherence [46]. In terms of study design, all were randomized controlled studies (RCTs). The studies were conducted on men and women between the ages of 18 and 60, with an average age of 48.3 years. On the other hand, seven studies included participants with low socioeconomic statuses, [40,41,43,44,46,47,48], one on the low-half socioeconomic level, and one on the half-high socioeconomic level (Table 2).

### 3.3. Characteristics of the Interventions

In terms of the type of exercise, two of the nine studies involved walking [41,47]. In one study, physical activity was performed in relation to work activity and leisure time [44], and in another study, graded aerobic, stretching, and strengthening exercises were performed [48]. Five studies did not describe the type of exercise [40,42,43,45,46].

In relation to mHealth, in six of the nine studies, the intervention groups received text messages and/or calls [40,41,42,43,44,45]; in another, education on exercise performance and text messaging [46]; in another article, voice messages [47]; and in another, a tutorial for a mobile app [48]. Regarding EMA (ecological momentary assessment), two of the nine studies used text messages and questionnaires [40,41]; one was a text-only study to collect information [45]; one study used a mobile phone app, calls [42], and another app only [48]; two studies used text messages and calls [43,47]; one study used text messaging and medical treatment reassessing risk factors [44]; and one study used text messaging and educational sessions [46]. Text messages were the most used alternative, included in six of the nine studies, and calls were included in three of the nine studies.

Regarding the dose–responses of physical exercise in the intervention, two of the nine selected studies presented records of the duration of the session: in one study, it was 20 min [41]; in the other, it was 30 min [40]. Two studies presented records of sessions per week: in one study, they occurred 5 days per week [40], and in the other, 3 days a week [41]. The nine selected studies presented records of the duration of the intervention in months, this characteristic being the most reported in the studies: in one study, the intervention was at least one and a half months in duration [48]; three studies were 12 months in duration, this being the most commonly used option in the studies [43,44,47]; and one study lasted 24 months [45]. Exercise intensity was recorded in only two studies: one study with low–moderate–high intensity [41] and the other with moderate intensity [44] (Table 3).

### 3.4. Study Variables and Outcomes

Table 4 shows the variables studied, the effects of the interventions, and the instruments used. Of the nine studies selected, in six studies, there was a positive effect on physical activity and/or quality of life [40,42,44,45,46,47]. In one study, there was a positive effect on quality of life when including physical activity [48], and two studies suggested no effect [41,43].

Regarding the nutrition variable, of the nine selected studies, there was a positive effect in six studies [40,41,42,43,45,47]. Another variable to highlight is weight: of the nine selected studies, there was a positive effect in three studies [42,43,44]. For the blood pressure variable, of the nine selected studies, there was a positive effect in three studies [40,44,46]. There was a positive effect on glucose in two studies [44,47]. There were positive effects in six of the nine studies on variables such as physical activity and nutrition, and in five of the nine studies on medical assessments, but for variables such as quality of life and anthropometric measurements, there were only positive effects in two of the nine studies; therefore, they were not significant in most studies. Regarding the nutrition variable, of the nine selected studies, there was a positive effect in six studies [40,41,42,43,45,47]. Another variable to highlight is weight: of the nine selected studies, there was a positive effect in three studies [42,43,44]. For the blood pressure variable, of the nine selected studies, there was a positive effect in three studies [40,44,46]. There was a positive effect on glucose in two studies [44,47]. There were positive effects in six of the nine studies on variables such as physical activity and nutrition, and in five of the nine studies on medical assessments, but for variables such as quality of life and anthropometric measurements, there were only positive effects in two of the nine studies; therefore, they were not significant in most studies.

On the other hand, among the most used instruments to measure the physical activity variable, different versions of physical activity questionnaires present in four of the nine selected studies stand out. For example, the IPAQ International Physical Activity Questionnaire was used in two studies [41,43]; in one study, the RPAQ Physical Activity Questionnaire [45]; and in another, the Global Physical Activity Questionnaire [46] (see Table 4).

### 3.5. Risk of Bias Assessment

**Figure 2:**
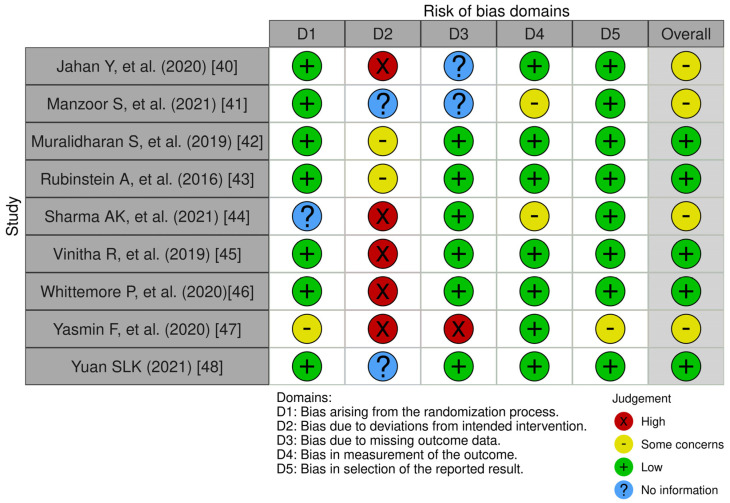
Traffic light plot of the risk of bias of the selected RCAs [39].

**Figure 3:**
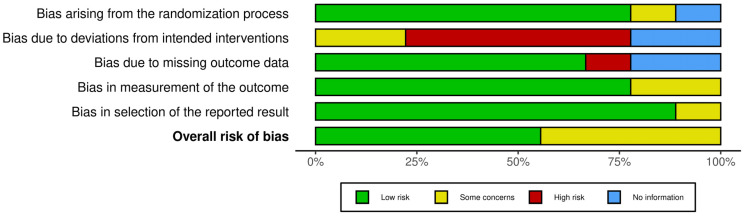
Summary plot of risk of bias.

The results of the nine randomized clinical trials included in this review are presented based on the domains proposed by the Cochrane manual. Risk of bias analysis revealed that the distribution of biases classified as “low risk or some concerns” was similar across the domains: (D1) selection bias, (D4) attrition risk, and (D5) reporting bias. In the detection bias domain (D3), articles were detected without sufficient information to make a conclusion regarding the bias (2/9). On the other hand, in the performance bias domain (D2), a high percentage of articles with “high risk” were detected (5/9) (Figure 2 and Figure 3).

## 4. Discussion

The aim of this study is to synthesize the methodological characteristics and health effects of physical exercise programs based on mobile health and ecological momentary assessment in adults in developing countries. The main findings of this review indicate that the selected studies were mostly conducted in South Asia, with the physical pathologies mainly being type 2 diabetes and overweight or obesity. Most of them were conducted in populations of low socioeconomic status. The mHealth interventions were mostly carried out through text messages, and only two used a mobile application; likewise, the follow-up with EMA was developed mainly with text messages, with calls as the second option. The interventions had durations per session between 20 and 80 min, 3 to 5 days a week, and were mostly applied for 12 months. In terms of exercise intensity, the most used options were low–moderate–high and moderate. In seven of nine studies, among the interventions with the most significant effects, the dose–response of the interventions yielded 30 min per session, 5 days a week, between a month and a half to 12 months of moderate duration and intensity. There was a significant effect in most studies on variables such as physical activity, nutrition, and medical assessments, but variables such as quality of life and anthropometric measurements were not significant in most studies.

### 4.1. Study Characteristics

Regarding the type of exercise used in the interventions, in our review, we found studies that performed walking; physical activity in relation to work activity and leisure time; aerobics; and stretching and strengthening graduate exercises, i.e., most used both aerobic and strengthening exercises. There were studies that did not describe the type of exercise.

These results are consistent with another study where the exercises were mainly divided into three main categories: aerobic exercises, strength exercises, and flexibility exercises. There were 102 different types of movement within the app that allowed exercise according to these three different features. In addition, the movements in the exercise sessions were ordered from easy to difficult, taking into account individual differences [49]. In line with recent results, in a study, the physical activity intervention component in addition to applying walking promoted increased daily steps, moderate-to-vigorous-intensity physical activity (MVPA), and resistance training (RT) activity to align with physical activity and sedentary behavior guidelines [50]. The use and combination of both aerobic and strength exercises have positive health implications, given that most of the activities of daily living, both at home and at work, involve aerobic capacity and strength, both of which represent important health indicators for both men and women. In the case of physical activity in relation to work activity and free time, it has an implicit component of a social nature, which leads participants to interact with their peers; there is greater equity in terms of improving aerobic capacity, strength, and flexibility through stretching exercises, especially for executive or office jobs in which physical activities are reduced.

In relation to the dose–response of physical exercise in the intervention, in our systematic review, all the selected studies presented records of the duration of the intervention in months. This characteristic was the most reported in the studies, with one study of at least 1 month and a half in duration and three studies of 12 months duration. This option was the most used in the studies. The duration of the intervention in months may be relevant, since a longer intervention would allow us to see the permanence and effectiveness of the variables studied. The duration of the sessions fluctuated between 20 and 30 min; the sessions per week varied between 3 and 5 days per week; and in terms of exercise intensity, the options used were low–moderate–high and moderate intensity. The use of different exercise intensities may be important in order to evaluate, examine, and calculate estimates of sedentary times and low-, moderate-, and high-intensity exercise.

In accordance with the results obtained in this review, it has been found in another study that a longer intervention may be more appropriate to determine whether adherence to the target behavior is maintained for a longer period of time [51]. As they are longer interventions, it allows for the permanence of the variables studied to be improved over time, including physical exercise, and increases the probability of establishing a behavior of performing physical exercise, which implies improvements in the health of adult men and women. In addition, by carrying out the interventions in a group, this improves the lives of adults from a social point of view by increasing interaction and creating new relationships between people.

These results are consistent with a study where physical activity was assessed using an Actigraph wGT3X-BT accelerometer-based device. Participants always wore the device on their hip, except during sleep and water activities (e.g., showering, swimming). They prepared summary files to examine the ratio with valid data (minimum acceptable time of use is 10 h/day with 4 valid days) eliminated outliers and calculated estimates of time spent on both sedentary behaviors and light, moderate, and vigorous physical activity [52]. Calculating estimates of time spent on sedentary behaviors and light, moderate, and vigorous physical activity is important for exercise prescription and intensity for adult men and women.

In line with the above, different data have been reported in another study regarding the control arm with which the intervention was to be compared, so it was designed as an active control. Patients assigned to the active control group received brief advice from their GPs, including a recommendation to self-monitor their daily steps and aim to reach a recommended target of an additional 3000 daily steps using a Fitbit tracker. Active control rather than passive control was chosen primarily so that we could isolate the net effect of the mHealth intervention. Complex interventions should be compared with a control consisting of self-monitoring and a set goal to demonstrate the additional benefits beyond self-control on its own. The choice of active control increases both the internal and external validity of the trial and is in line with the pragmatic design of the study [53]. Having an active control group in addition to the experimental group increases the number of people with health benefits and implies greater equity in adult men and women in terms of the results of the variables studied. The number of people who improve their physical condition is increasing, which allows them to be in a better condition for the physical activities of daily living, as well as domestic chores.

### 4.2. Characteristics of mHealth and MEA Interventions

With respect to mHealth, in six of the nine studies selected from this systematic review, the intervention groups received text messages and/or calls, this being the most used option. Although other studies have reported data that are not consistent with the results of this review, the use of text messages and/or calls is a more common alternative in developing countries. This may be due to less education on the use of apps and less digital use, although, regarding the use of mHealth in developed countries, it is mentioned that a growing body of evidence has been established on behavior changes using personalized mHealth apps (apps) and their effective role in changing lifestyle risk factors. Therefore, theoretically based behavior change interventions using mHealth apps could provide researchers and others with an effective way to improve physical activity- and healthy lifestyle-related behaviors [51]. The use of applications saves time and money in locomotion and travel; therefore, it represents a good alternative for the practice of physical exercise.

In line with the above, different data have been reported with respect to another study where, in addition to receiving notifications, participants in the intervention arm had access to a mobile study app that paired with their smartwatch. The mobile app allowed for self-monitoring of activity data (step count, exercise minutes) and allowed participants to set and complete activity goals, which could be adjusted based on performance [54]. This facilitated not only the fulfillment of physical exercise goals, but also an improvement in physical fitness and health for both adult men and women. The self-monitoring and combination with their smartwatches allow them to monitor all the physical exercise and activity for the day.

In this regard, another study pointed out that mHealth programs could help to lessen the time spent on sedentary behavior. Mobile health interventions at work are viable, acceptable, and effective tools to promote physical activity and reduce sedentary lifestyles, even outside of working hours. However, studies evaluating the impact of these specific, long-term mHealth programs on occupational sedentary behavior are few. mHealth interventions, as a means of self-management and health promotion, offer a promising solution in primary health care to address the growing demand for treatment and control of T2DM. In addition, the use and effectiveness of mHealth programs are mostly focused on healthy adults, without considering the impact they may have on specific population groups, such as workers with prevalent chronic diseases such as obesity or T2DM. There is a need for a better understanding of how to integrate mHealth programs into the self-management of patients with chronic diseases, specifically T2DM self-management, and health care in general [55].

Regarding EMA, in this review, some studies used text messages and questionnaires, and another study only text messages to collect information. One study used a mobile phone app and calls, and another used only an app. Other studies used text messages and calls, one study used text messages and risk factors for reassessing medical treatment, and one study used text messaging and educational sessions. The most used alternatives in studies in developing countries were text messaging and, as a second choice, calls. There are few physical exercise studies that, in addition to using mobile health, have an ecological momentary assessment, which allows for evaluation and feedback during the intervention process to improve health in adults in developing countries.

In agreement with the results obtained in the present review, another article indicates that text messages or emails tailored to the information a subject provided were sent, or education and coaching calls personalized to their health and nutrition status were offered. In some cases, smartphone apps and additional web-based education were provided, and devices were offered to verify conference-style education and steps forward, meaning greater options for participants [33]. The ecological momentary assessment by text messages or calls allowed us to know the real states of the people during the intervention; there was feedback that implied health improvements in both men and women. This process can be transferred to people’s daily lives by engaging in physical exercise as a way of self-evaluating their sensations over a period of time.

In line with the above, different data have been reported with respect to an article indicating that the secondary outcomes of PA (physical activity) and body weight were self-reported in min/week and assessed through EMA during a 12-month program. Hypothetical mediators included affective response to PA, perceived effort, and perceived autonomy. The EMA was delivered via the mobile app. Participants used the Catalyst mobile app to complete daily wake-up reports and record any exercise sessions during the full 12-month study. Participants responded to random reports for a total of 12 weeks, including the first 4 weeks of enrollment and the 4 weeks leading up to months 6 and 12. The EMA protocol included nine EMA reports, representing a combination of events and timing. Participants completed five types of event-based reports and four time-based sampling reports. Through EMA, affective response, perceived autonomy, and PA behavior were evaluated, indicating that the EMA was not only being applied, but that there was monitoring, recording, and evaluation of different areas in different time periods during the full 12-month study [56]. Ecological momentary evaluation through a mobile application can include different aspects. It allows for registration, evaluation, and monitoring, and has a more direct use allowing people to know their real state during the intervention, meaning a greater knowledge of the process, which implies health improvements in both men and women. This process, through mobile applications, can be transferred to people’s daily lives when they wish to perform a period of physical exercise that allows them to self-evaluate their sensations over a period of time.

Regarding EMA, although mobile devices are important as a method of supervised exercise in mobile health, where factors such as the adherence of the supervised persons to the plan and the methods used for exercise are determinants in the effectiveness of the exercise, these mobile devices also play a fundamental role in providing information feedback during the intervention process in the EMA. In this regard, in the 2010s, the market for both smartphones and mobile applications expanded, giving way to the evaluation, monitoring, and follow-up of clinical activities [57]. In line with the above, other studies have reported that among the different measurement facets that the EMA can provide simultaneously are recording the place where one is in real time (home, school, park, stores, etc.); knowing who one is sharing the momentary experience with (alone, with friends, family, colleagues, etc.); and the feeling that the environment provides (security, tension, tranquility, pleasure). Likewise, it allows for the measurement of both positive and negative affective states [58,59,60,61].

### 4.3. Strengths and Limitations

The strength of this systematic review is the search for scientific literature that delves into the use of mHealth and EMA technology, with physical exercise interventions carried out in developing countries. Another strength is that it evidences studies conducted in vulnerable populations, given that most studies described a low socioeconomic level.

One limitation is differences in measurement instruments, samples, and different ways of collecting information in studies using mHealth in EMA mode with exercise interventions, making it difficult to analyze and interpret. Another limitation is that there are few studies evaluating the impact of specific mHealth programs at work and in the long term on occupational sedentary behavior. On the other hand, we observed that there was high heterogeneity in the methodological quality of the studies. The highest risk of bias reported by the Cochrane tool was related to performance bias and detection bias. Both biases were associated with difficulty in masking participants and study staff. These biases increased the risk that the knowledge of the intervention received would affect the results, which could lead to a systematic error in the application of EMA. Future researchers are invited to delve deeper into work-specific and more temporary mobile health interventions.

## 5. Conclusions

This systematic review included randomized controlled trials from different developing countries with respect to the sample, in which the most common diseases in adults were diabetes, overweight and obesity, and hypertension. A higher proportion of women was observed, and most studies fluctuated between 3 and 12 months in duration, with 12-month interventions being the most used option as a response to a study indicating that a longer intervention might be more appropriate to determine whether adherence to the target behavior was maintained for a longer period of time [51]. In all studies, mobile devices were used as the technology. The results of variables such as physical activity, nutrition, and medical evaluations had significant effects, and variables such as quality of life and anthropometric measurements were not significant in most studies.

It is suggested that the use of mHealth in EMA modality in physical exercise interventions for health in adults may be applicable to populations in different developing countries, vulnerable populations, and different health systems.

The results of this systematic review support the use of EMA modality mHealth interventions in physical exercise for health and may improve the medical prognoses of adult patients with cardiometabolic diseases.

## Figures and Tables

**Table 1:** Inclusion criteria.

Criteria	Description
(1) Population	Studies that included adults, men and women between 18 and 60 years old, with any health condition (healthy and with pathologies) who live in developing countries according to the classification established by the Organization for Cooperation and Development Economic (OCDE) [36] and developing countries in the WTO (World Trade Organization) [37].
(2) Intervention	Studies that used interventions involving physical exercise programs carried out with mHealth in EMA modality for their prescription.
(3) Outcome variables	Studies that evaluated at least one of the following variables: (a) Physical and metabolic health (physical condition, anthropometric measurements, nutrition, medical variables, daily energy expenditure, muscle mass, oxygen consumption, cardiorespiratory and metabolic benefits, lipid profile, glucose homeostasis, etc.); (b) Mental health (quality of life, self-esteem, well-being, mood, stress, anguish, depression, etc.); (c) Cognition (self-care, cognitive functions, memory, attention, etc.); (d) Social (social integration and other related).
(4) Study design	All articles with randomized controlled experimental designs which provide original data on the use of mHealth and EMA in physical exercise (criteria 1, 2 and 3) and are published in scientific journals.

**Note:** All published articles such as reviews, editorial documents, protocols, theses, conference abstracts, and letters to the editor were excluded.

**Table 2:** General characteristics of the included studies.

Author (Year), Country	Population					Intervention Group	Control Group	Outcomes	Design Type
	Health Condition and Sample I/F (n°)	Gender M/F	(n°) ADH	(%) Age	(M) NSE				
**Jahan Y, et al. (2020), Bangladesh. [40]**	Adults with hypertension 420/4,12	59/361	72.7	52.9	Low	The intervention group received SMS text messages and 5 months of health education along with a health education booklet. Text messages (SMS) were sent 5 times during the first month and once a week for the remaining 4 months (a total of 21 SMS text messages), along with questionnaires according to the DASH (Dietary Approaches to Stop Hypertensive) diet.	The control group received only face-to-face health education and not SMS text messages. They received the same health education pamphlet as the intervention group and were followed every month for up to 5 months (twice in the first month and once in the rest), but did not receive SMS text messages.	Physical activity Body weight Salt intake Fruit intake Vegetable intake Blood pressure Quality of life	Two-arm Randomized Controlled Study
**Manzoor S, et al. (2021), Pakistan. [41]**	Adults with coronary syndrome, 160/119	126/34	74.4	52.6	Low	First phase of Mcard included individualized counseling; second phase, daytime mobile texting of standardized messages using an app especially and standard post-ACS care. (Counseling, short text messages and standard post-ACS care). Healthy eating (healthy eating assessment tool) and physical activity (IPAQ tool).	Standard care after myocardial infarction Standard care after myocardial infarction	Physical activity Healthy nutrition Measurement compliance Smoking Salt intake Blood pressure Body weight	Two-arm Randomized Controlled Study
**Muralidharan S, et al. (2019), India. [42]**	Adults at high risk of DM2 (pre-DM and/or obesity), 741/561	320/241	-	37.8	Low, half	(mDiab) mobile health and diabetes program. It included video lessons, text messages, interactive text, and weekly coaching via calls and emails. The intervention was delivered via a mobile phone application along with weekly coach calls.	Control group received usual care Usual care	Physical activity Weight loss Diet	Randomized Controlled Three-armed study.
							Reading and carrying out guidelines on daily care based on mobility and healthy eating. Periodic messages (does not specify time).		
**Rubinstein A, et al. (2016) Argentina, Guatemala, Perú. [43]**	Adults with prehypertension. 637/553	295/342	84	43.6	Low	Monthly motivational coaching calls and weekly personalized text messages on diet quality and physical activity based on the transtheoretical model. Monthly motivational coaching calls and weekly personalized text messages.	Usual care Usual care	Physical activity Body weight Fruits and vegetables Less intake of foods rich in sodium, fat, and sugar Blood pressure Waist circumference	Two-arm Randomized Controlled Study
**Sharma AK, et al. (2021) Jaipur India. [44]**	Adults with metabolic syndrome 1200/1012	468/544	60	40	Low	mHealth group mobile health intervention in the form of daily one-way IVRS (interactive voice response system) audio clip in Hindi using a full automated system. Two messages promoting a healthy lifestyle and medical treatment were broadcast daily for 12 months and risk factors were reassessed.	Control group (standard care), provided by the local state government health center.	Physical activity Healthy diet Tobacco Waist circumference BMI Systolic pressure Fasting glucose Cholesterol	Two-arm Randomized Controlled Study
**Vinitha R, et al. (2019) India. [45]**	Adults with newly diagnosed type 2 diabetes. 248/218	168/80	-	43.3	Half, high.	The recent physical activity questionnaire (RPAQ), A.F, was performed for the last 4 weeks, and sleep duration was also used. European Quality of Life Questionnaire 5 Dimension (EQ5D), 24 h recall diet, and text message acceptability questionnaires were completed at 6, 8, 12, and 24 months. They were counseled on an individual level about healthy behavioral changes to follow a pattern of a healthy diet and physical activity, and they also received 2 to 3 personalized educational text messages per week until the end of the study.	Control group, standard care advice only at the beginning. Standard.	Blood pressure glycemia LDL HbA1c	Two-arm Randomized Controlled Study
**Whittemore P, et al. (2020) México. [46]**	Low-income adults with type 2 diabetes. 85/82	58/27	89	55.5	Low	DSME (Yes, I can!) program (diabetes self-management education program plus text message). It consisted of 7 weekly interactive group educational sessions on diabetes self-management and 6 months of daily texting/pictures. Physical activity was measured with the global physical activity questionnaire, which consists of 16 items that evaluate the frequency, intensity, and duration carried out at work and during transportation and free time, as well as sedentary behavior.	Standard DM2. Standard diabetes care		Two-arm Randomized Controlled Study
**Yasmin F, et al. (2020) Dhaka Bangladesh. [47]**	Adults with type 2 diabetes, 320/273	71/249	-	52	Low	Intervention group included mobile phone reminders and a 24/7 call center. Reminder system through interactive voice calls one or two days before scheduled hospital visits, a related information and suggestion service, and a 24/7 call center service. Average duration of each call was 10 min.	They only received regular hospital services. They only received regular hospital services.	Physical exercise Diet Tobacco and betel nut consumption Blood glucose	Two-arm Randomized Controlled Study
**Yuan SLK, Couto LA, AP Trademarks (2021) Brasil. [48]**	Adults with fibromyalgia, 40/36	1/39	40	43.3	Low	ProFibro App Application Group (PAG). Program with aerobic, stretching, and strengthening exercises. Evaluated through the ProFibro App (PAG), participants received a 20-minute tutorial that guided them through the app’s features or book chapters.	Paper Book Group (PBG), a traditional paper book group with similar content, symptoms, and self-care agency for patients. 20-minute introductory tutorial that guided participants through app features or book chapters.	Physical activity Pain Quality of life	Two-arm Randomized Controlled Study

**-**: No information; ADH: adhesion; NSE: socioeconomic; IPAQ: International Physical Activity Questionnaire; RPAQ: Recent Physical Activity Questionnaire; EQ5D: European Quality Questionnaire 5 Dimension, DM2: diabetes mellitus Type 2; HbA1c: glycosylated hemoglobin.

**Table 3:** General characteristics of the included studies.

Author (Year), Country	Groups	Frequency	Exercise INTENSITY	Session Duration (Min)	Sessions per Day	Intervention Duration	Type of Exercise
		Weekly Sessions			(n°)	(Months)	
**Jahan Y, et al. (2020), Bangladesh. [40]**	IG	5	-	30	-	5 months	-
	CG	-	-	-	-	-	
**Manzoor S, et al. (2021), Pakistan. [41]**	IG	3	Low, moderate, high.	20	-	6 months	Hike
	CG	-	-	-	-	-	
**Muralidharan S, et al. (2019), India. [42]**	IG	-	-	-	-	3 months	They encouraged physical activity; the type of exercise, intensity and duration was not specified.
	CG1	-	-	-	-	-	
	CG2	-	-	-	-	-	
**Rubinstein A, et al. (2016) Argentina, Guatemala, Peru. [43]**	IG	-	-	-	-	12 months	-
	CG	-	-	-	-	-	-
**Sharma AK, et al. (2021) Jaipur India. [44]**	IG	-	Moderate	-	-	12 months	Physical activity in relation to work activity and free time activity.
	CG	-	-	-	-	-	-
**Vinitha R, et al. (2019) India. [45]**	IG	-	-	-	-	24 months	-
	CG	-	-	-	-	-	-
**Whittemore P, et al. (2020) México. [46]**	IG	-	-	-	-	6 months	-
	CG	-	-	-	-	-	-
**Yasmin F, et al. (2020) Dhaka Bangladesh. [47]**	IG	5	-	30 min	1	12 months	The patient was considered adherent if he walked 30 min per day or 150 min in a week (according to the WHO).
	CG	-	-	-	-	-	-
**Yuan SLK, Couto LA, AP Trademarks (2021) Brasil. [48]**	IG	-	-	-	-	1 month and a half	Graduated aerobic, stretching, and strengthening exercise program.
	CG	-	-	-	-	-	-

**Note: -**: No information; IG: intervention group; CG: control group; IPAQ: International Physical Activity Questionnaire; WHO: World Health Organization.

**Table 4:** Effect of physical exercise based on mHealth in MEA modality.

		Physical and Metabolic Health				Mental Health	Cognition	Social
Author (Year)	Groups	Physical Activity	Anthropometric Measurements	Nutrition	Medical Variables	Quality of Life		
**Jahan Y, et al. (2020), Bangladesh. [40]**	IG	*	Body weight monitoring *	Salt intake * Fruit intake * Vegetable intake *	Blood pressure monitoring *	Quality of life *	-	-
	CG	*	Body weight monitoring *	Salt intake * Fruit intake * Vegetable intake *	Blood pressure monitoring *	Quality of life *	-	-
**Manzoor S, et al. (2021), Pakistan. [41]**	IG	*	Weight =	Healthy nutrition * Salt consumption * Smoking *	Self-monitoring of BP * Medication compliance *	-	-	-
	CG	-	-	Healthy nutrition *	-	-	-	-
**Muralidharan S, et al. (2019), India. [42]**	IG	*	Weight *	Diet *	-	-	-	-
	CG1	-	Weight =	-	-	-	-	-
	CG2	-	Weight =	-	-	-	-	-
**Rubinstein A, et al. (2016) Argentina, Guatemala, Perú. [43]**	IG	=	Body weight * Body Mass Index (BMI). * Waist circumference =	Improvement in diet quality * Fruits and vegetables * Lower intake of foods rich in sodium and fat *	Systolic Pressure = Diastolic Pressure = Net Pressure =	-	-	-
	GC	-	-	-	Blood pressure *	-	-	-
**Sharma AK, et al. (2021) Jaipur India. [44]**	IG	*	Weight * Waist circumference (HC). * Body Mass Index (BMI). *	Healthy diet * Tobacco *	Blood pressure * Fasting glucose * Total cholesterol * Triglycerides * HDL Cholesterol * LDL Cholesterol *	-	-	-
	CG	*	Weight * Waist circumference (HC). * Body Mass Index (BMI). *	Healthy diet * Tobacco *	Blood pressure * Fasting glucose * Total cholesterol * Triglycerides * HDL Cholesterol * LDL Cholesterol *	-	-	-
**Vinitha R, et al. (2019) India. [45]**	IG	*	-	Diet *	Blood pressure * Blood glucose * LDL Cholesterol * Glycosylated hemoglobin (HbA1c) *	Quality of life *	-	-
	CG	-	-	-	Blood pressure * Blood glucose * LDL Cholesterol * Glycosylated hemoglobin (HbA1c) *	-	-	-
**Whittemore P, et al. (2020) México. [46]**	IG	*	-	-	Systolic and diastolic BP * Glycosylated hemoglobin (HbA1c) *	Depressive symptoms * Self-efficacy *	-	-
	CG	-	-	-	-	-	-	-
**Yasmin F, et al. (2020) Dhaka Bangladesh. [47]**	IG	*	-	Diet (CHO, proteins, fats). * Intake of fruits and vegetables * Smokeless tobacco, chewing tobacco and betel nut * Tobacco or cigarette smoke *	Fasting blood glucose level * Blood glucose level 2 h after breakfast *	-	-	-
	CG	=	-	Diet (CHO, proteins, fats). = Intake of fruits and vegetables = Smokeless tobacco, chewing tobacco, and betel nut = Tobacco or cigarette smoke =	Fasting blood glucose level = Blood glucose level 2 h after breakfast =	-	-	-
**Yuan SLK (2021) [48]**	IG	*	-	-	Pain * Severity of symptoms *	Quality of life *	Self-care *	-
	CG	*	-	-	Pain * Severity of symptoms *	Quality of life *	Self-care *	-

**-**: No information; *: there were significant effects; =: there were no effects. BMI: Body Mass Index; BP: blood pressure; HbA1c: glycosylated hemoglobin; CHO: carbohydrates.

## Data Availability

All data are available in the reference list of the article selected for this review.

## Supplementary Materials

The following supporting information can be downloaded at: https://www.mdpi.com/article/10.3390/medicina60040578/s1, The PRISMA checklist.
