# Supplementary material for: Effect of Physical Exercise Programs Based on Mobile Health and Ecological Momentary Assessment on the Physical and Mental Health, Cognitive Functions, and Social Environment of Adults in Developing Countries: A Systematic Review
Source: Medicina (Kaunas). 2024 Mar 31;60(4):578. doi: 10.3390/medicina60040578 (PMC11052404; doi:10.3390/medicina60040578)
Supplement: Supplementary file 1 [file medicina-60-00578-s001.zip › medicina-2889255-supplementary.pdf]

Effects of physical exercise interventions through Mobile Health and Ecological Momentary Assessment on physical, metabolic, and psychological health in adults, in developing countries: A systematic review.

To enable PROSPERO to focus on COVID-19 submissions, this registration record has undergone basic automated checks for eligibility and is published exactly as submitted. PROSPERO has never provided peer review, and usual checking by the PROSPERO team does not endorse content. Therefore, automatically published records should be treated as any other PROSPERO registration. Further detail is provided here.

Citation

Alejandro Flores Aniotz, Margarita Rubio Alonso, Sonia García Merino, Margarita Pérez Ruíz, Igor Cigarroa Cuevas, Víctor Contreras Mellado., Rafael Zapata-Lamana. Effects of physical exercise interventions through Mobile Health and Ecological Momentary Assessment on physical, metabolic, and psychological health in adults, in developing countries: A systematic review.. PROSPERO 2022 CRD42022347874 Available from: https://www.crd.york.ac.uk/prospero/display_record.php?ID=CRD42022347874

Review question

What are the effects of physical exercise interventions through the use of Mobile Health and Ecological Momentary Assessment on physical, metabolic, and psychological health in adults, in developing countries?

What are the methodological characteristics of studies that use Mobile Health and Ecological Momentary Assessment on adult health in developing countries?

Searches

The following databases will be used: Central de Cochrane, MEDLINE de PubMed, Core Collection de Web of Science, PsycINFO de PsycNET, CINAHL de EBSCOhost, SciELO, LILACS.

The selected articles were published between 2011 and 2022, period in which the use of mHealth technology greatly improved in developing countries. Moreover, the search will be defined by population (human beings), and English and Spanish language.

Types of study to be included

Random controlled tests that assess the effects of the health programs that incorporate the use of mHealth and EMA will be included. Different study designs will not be included.

Condition or domain being studied

The health field in which the review is focused corresponds to prevention and promotion of health programs in primary healthcare centers. These attend to healthy adults with risk factors such as low levels of physical condition, especially cardiorespiratory capacity and muscular strength, as well as metabolic risk factors, such as levels of glycemia and lipid profile, blood pressure, weight and BMI, and psychological risk factors, such as well-being and quality of life.

Participants/population

Inclusion: Adults; men and women between 18 and 59 years old, healthy or with risk factors of noncommunicable

chronic diseases such as diabetes, hypertension and overweight, who live in developing countries according to OECD.

Exclusion: People with musculoskeletal pathologies, such as dislocation or prosthetics that restrict their ability to exercise, and people with pathologies like dementia, which difficult understanding of instructions to exercise are excluded.

Intervention(s), exposure(s)

The review will focus on delving into the effects of physical exercise-based interventions, which have used mobile health (mHealth) technology, and Ecological Momentary Assessment (EMA), on the health of adult population of developing countries.

The effects of exercise on integral (physical, metabolic, and psychologic) health. Furthermore, the methodological aspects of articles will be studied: samples, duration of the studies, technology and interventions applied.

Comparator(s)/control

Group of adults; men and women between 18 and 59 years old, with risk factors such as low levels of physical condition, especially cardiorespiratory capacity and muscular strength, as well as metabolic risk factors, such as levels of glycemia and lipid profile, blood pressure, weight and BMI, and psychological risk factors, such as well-being and quality of life, who participate in physical exercise interventions in an in-person manner.

Context

Only research including the study of population from low- and middle-income developing countries, according to the OECD classification will be included.

Main outcome(s)

Physical health: improvement on the physical condition related to health, specifically cardiorespiratory capacity and muscular strength through physical exercise. The cardiorespiratory capacity will be evaluated by means of direct and indirect tests, and muscular strength of upper limbs through manual pressure dynamometry.

Metabolic health: improvement on cardiometabolic variables, such as levels of glycemia and lipid profile, which will be evaluated by means of a blood test. Blood pressure will be measured with a sphygmomanometer or a digital tensiometer. Weight and BMI will be measured with traditionally standardized protocols.

Psychological health: Improvement on well-being and quality of life and associated variables through physical exercise, measured through self-report in real time.

Additional outcome(s)

Participation in the physical exercise intervention sessions will be evaluated.

Data extraction (selection and coding)

Firstly, duplicated articles will be deleted from the database by means of a bibliographic manager. Then, two reviewers will apply inclusion/exclusion criteria to all the titles and abstracts. Those articles matching the inclusion criteria will be selected, and when classification is not possible based solely on the title and abstract, the entire article will be retrieved.

The chosen articles will be reviewed independently by two reviewers. Discrepancies will be solved through a discussion with a third author if needed, until consensus is reached.

A standardized previously tested form will be used to extract the data from the included articles in order to assess quality of the study, and to synthetize the evidence.

Risk of bias (quality) assessment

Cochrane risk-of-bias tool will be used to assess bias in randomized trials.

Two independent reviewers will analyze the methodological quality of the selected studies.

Strategy for data synthesis

The data extracted from the articles will include: general information (author, year, country), sample (size, population, age group, etc.), intervention characteristics and its effects on health, details about mHealth and EMA tools (measure, objective/use, type of tool, etc.), methodological protocol (experimental design, response rates, time needed for the application, administration mode, use of sensors, etc.), as well as appraisal of the main variables. Moreover, the extraction of data will be carried out by two independent reviewers. The results will be presented in tables and the information analysis will be quantitative.

Analysis of subgroups or subsets

Not applicable

Contact details for further information

Rafael Zapata-Lamana

rafaelzapata@udec.cl

Organisational affiliation of the review

None

Review team members and their organisational affiliations

Ms Alejandro Flores Aniotz. Vicerrectoría de Pregrado, Programa Vida Saludable, Universidad de Talca

Dr Margarita Rubio Alonso. Departamento de Medicina, Facultad de Ciencias Biomédicas y de la salud, Universidad Europea de Madrid

Dr Sonia García Merino. Universidad Francisco de Vitoria

Dr Margarita Pérez Ruíz. Departamento Salud y Rendimiento Humano Facultad de Ciencias de la Actividad Física y el Deporte-INEF. Universidad Politécnica

Dr Igor Cigarroa Cuevas. Escuela de Kinesiología, Facultad de Salud, Universidad Santo Tomás, Santiago

Víctor Contreras Mellado.. Vicerrectoría de Pregrado, Programa Vida Saludable, Universidad de Talca, Chile.

Dr Rafael Zapata-Lamana. Centro de vida saludable, Universidad de Concepción, Chile

Collaborators

Dr Natalia Ulloa. Centro de vida saludable Universidad de Concepción

Type and method of review

Individual patient data (IPD) meta-analysis, Systematic review

Anticipated or actual start date

02 May 2022

Anticipated completion date

23 March 2023

Funding sources/sponsors

“Ejercicio físico vía Mobile- Health: Un desarrollo innovador”. Project financed by Fondo de Innovación para la Competitividad, regional government of Biobío, and implemented by Universidad de Concepción, Chile (código:40026768-0).

Conflicts of interest

Language

English

Country

Chile

Stage of review

Review Ongoing

Subject index terms status

Subject indexing assigned by CRD

Subject index terms

Adult; Developing Countries; Ecological Momentary Assessment; Exercise; Exercise Therapy; Humans; Telemedicine

Date of registration in PROSPERO

31 July 2022

Date of first submission

20 July 2022

Details of any existing review of the same topic by the same authors

Stage of review at time of this submission

The review has not started

Preliminary searches	No	No
Piloting of the study selection process	No	No
Formal screening of search results against eligibility criteria	No	No
Data extraction	No	No
Risk of bias (quality) assessment	No	No
Data analysis	No	No

The record owner confirms that the information they have supplied for this submission is accurate and complete and they understand that deliberate provision of inaccurate information or omission of data may be construed as scientific misconduct.

The record owner confirms that they will update the status of the review when it is completed and will add publication details in due course.

Versions

31 July 2022

31 July 2022
